# Phosphorylation of Rat Melanopsin at Ser-381 and Ser-398 by Light/Dark and Its Importance for Intrinsically Photosensitive Ganglion Cells (ipRGCs) Cellular Ca^2+^ Signaling*

**DOI:** 10.1074/jbc.M114.586529

**Published:** 2014-11-06

**Authors:** Jan Fahrenkrug, Birgitte Falktoft, Birgitte Georg, Jens Hannibal, Sarah B. Kristiansen, Thomas K. Klausen

**Affiliations:** From the ‡ Department of Clinical Biochemistry, Faculty of Health Sciences, Bispebjerg Hospital, DK-2400 Copenhagen NV, Denmark and; § Department of Biology, Faculty of Science, University of Copenhagen, DK-2200 Copenhagen N, Denmark

**Keywords:** Calcium Imaging, G Protein-coupled Receptor (GPCR), Mutant, Phosphorylation, Photoreceptor, Retinal Ganglion Cells

## Abstract

The G protein-coupled light-sensitive receptor melanopsin is involved in non-image-forming light responses including circadian timing. The predicted secondary structure of melanopsin indicates a long cytoplasmic tail with many potential phosphorylation sites. Using bioinformatics, we identified a number of amino acids with a high probability of being phosphorylated. We generated antibodies against melanopsin phosphorylated at Ser-381 and Ser-398, respectively. The antibody specificity was verified by immunoblotting and immunohistochemical staining of HEK-293 cells expressing rat melanopsin mutated in Ser-381 or Ser-398. Using the antibody recognizing phospho-Ser-381 melanopsin, we demonstrated by immunoblotting and immunohistochemical staining in HEK-293 cells expressing rat melanopsin that the receptor is phosphorylated in this position during the dark and dephosphorylated when light is turned on. On the contrary, we found that melanopsin at Ser-398 was unphosphorylated in the dark and became phosphorylated after light stimulation. The light-induced changes in phosphorylation at both Ser-381 and Ser-398 were rapid and lasted throughout the 4-h experimental period. Furthermore, phosphorylation at Ser-381 and Ser-398 was independent of each other. The changes in phosphorylation were confirmed *in vivo* by immunohistochemical staining of rat retinas during light and dark. We further demonstrated that mutation of Ser-381 and Ser-398 in melanopsin-expressing HEK-293 cells affected the light-induced Ca^2+^ response, which was significantly reduced as compared with wild type. Examining the light-evoked Ca^2+^ response in a melanopsin Ser-381 plus Ser-398 double mutant provided evidence that the phosphorylation events were independent.

## Teaser

**Background:** Melanopsin is a retinal light-sensitive receptor involved in non-image forming functions including circadian timing.

**Results:** The intracellular C-terminal part of melanopsin was shown to be phosphorylated at distinct functional sites in response to light and dark.

**Conclusion:** Phosphorylation status of melanopsin influences its functions.

**Significance:** Distinct sites of phosphorylation are important in determining the signaling properties of melanopsin.

## Introduction

Melanopsin is a unique light-sensitive receptor that is expressed in the membrane of a subset of retinal ganglion cells of the mammalian eye, rendering these cells intrinsically photosensitive. These intrinsically photosensitive ganglion cells (ipRGCs),^2^ which also receive input from the rods and cones, constitute a non-image photoperception system measuring environmental light intensities. The ipRGCs, which project to the brain biological clock in the hypothalamic suprachiasmatic nucleus and other brain areas, are involved in the regulation of circadian timing, masking behavior, melatonin secretion, and the pupillary light reflex (1). In the mouse retina the ipRGCs are classified in five different subtypes according to morphology, stratification, and light response (1–4).

The melanopsin-expressing cells respond to light stimulation with an absorption maximum around 480 nm, which elicits a sluggish depolarization of the membrane potential (5), an increase in intracellular Ca^2+^ (6–8), and a sustained expression of the protein encoded by the immediate early response gene *Fos* (9, 10). Melanopsin is both *N*-link- and *O*-link-glycosylated, but glycosylation does not seem to be crucial for the melanopsin response to light (11). The amino acid sequence of mammalian melanopsin is consistent with the protein being a G protein-coupled receptor, and there is growing evidence that melanopsin activates a Gα_q/11_ G protein followed by stimulation of phospholipase C, which leads to the opening of cation-selective transient receptor potential ion channels (TRPC) (1). Extracellular signal-regulated protein kinases 1 and 2 (ERK1/2) seem to be essential for the expression of *Fos* upon illumination (12). Activation of G protein-coupled receptors are often followed by phosphorylation at Ser and/or Thr residues of the C-terminal receptor tail, which could be involved in intracellular signaling and receptor trafficking (13). Thus, it is likely that phosphorylation of melanopsin upon light activation takes place and that phosphorylation could be important for the regulation of melanopsin function. Recently, the first evidence was provided that mouse melanopsin is phosphorylated in the C-terminal tail in a light-dependent manner (14) and subsequently a cluster of Ser and Thr residues in the region between amino acid 386 to 396 was shown to be involved in mediating deactivation upon light stimulation (15). However, the specific phosphorylation sites of the C-terminal tail of melanopsin are yet to be identified.

In the present study we first used bioinformatics to identify a number of high probability phosphorylation sites in the long C-terminal cytoplasmic tail of rat melanopsin. On the basis of this, we generated phospho-site-specific antibodies against Ser-381 and Ser-398 and characterized them by immunoblotting and immunocytochemistry. The antisera were used to show light-induced changes in phosphorylation at these sites both *in vitro* and *in vivo*. Furthermore, we demonstrated that elimination of phosphorylation by Ser to Ala mutations at these sites significantly reduced the intracellular Ca^2+^ response upon light stimulation.

## EXPERIMENTAL PROCEDURES

### 

#### 

##### Antisera

**FIGURE 1.**
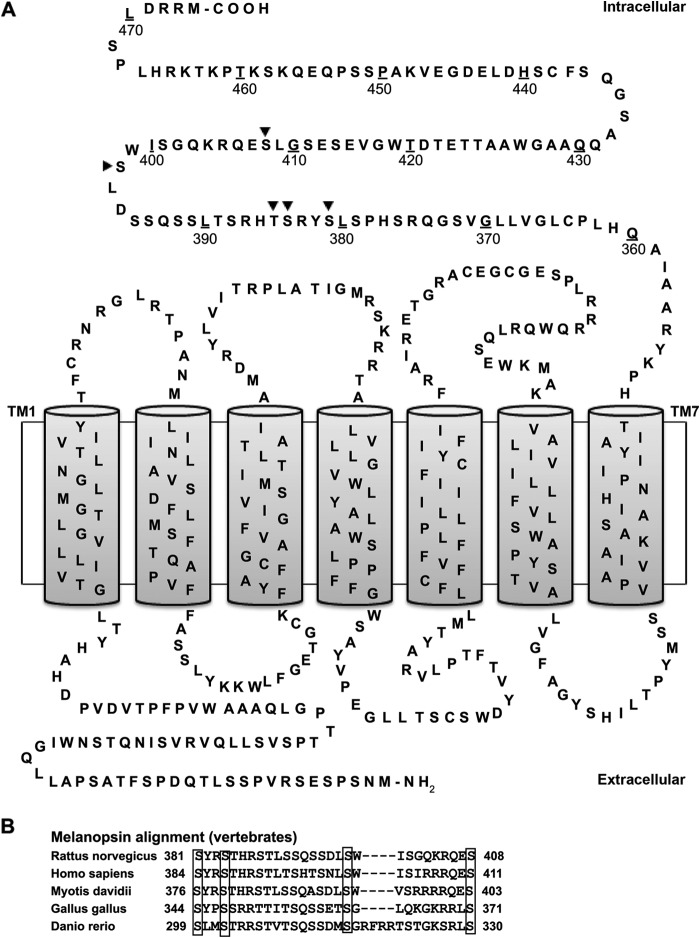
*A*, secondary structure of rat melanopsin. Predicted transmembrane domains (*TM*) are indicated by *shaded columns*. Amino acid residues in the C-terminal intracellular domain with high probability of being phosphorylated according to their NetPhos score are indicated by *arrowheads*. The *numbers* indicate the amino acid positions in the receptor protein. *B,* alignment of amino acid sequences of melanopsin of species belonging to different classes of vertebrates and orders of mammals illustrating that four of the potential phosphorylation sites shown in *panel A* are conserved among these. The conserved residues are indicated by *boxes*, and the following accession numbers were used for the alignment: *Rattus norvegicus* (NP_620215), *Homo sapiens* (NP_150598), *Myotis davidii* (ELK27115), *Gallus gallus* (NP_001038118), and *Danio rerio* (NP_001122233).

The amino acid sequence of rat melanopsin (NP_620215.1 from the NCBI Protein Data Bank) was analyzed by the bioinformatics web-based tool NetPhos 2.0, which scans protein sequences and calculates a theoretical probability of an amino acid residue to be phosphorylated (16). Using the NetPhos 2.0 server at The Technical University of Denmark, four serines (Ser-381, Ser-384, Ser-398, and Ser-408) and one threonine (Thr-385) in the intracellular C-terminal tail of melanopsin (Fig. 1*A*) showed high phosphorylation potential (prediction score 0.93–0.99). Four of these were by sequence homology found to be conserved in mammalian and other vertebrate species (Fig. 1*B*). Custom phospho-site-specific antibodies against rat melanopsin phosphorylated at Ser-381 (abMel-P381) or Ser-398 (abMel-P398) were subsequently produced by Pacific Immunology Corp., Ramona, CA. The sequences of the two peptides containing the phosphorylated version of either Ser-381 and or Ser-398 were C-RSHPSLpSYRSTHRSTLS and C-SSQSSDLpSWISGQKR (pS is phosphoserine), respectively. The peptides were conjugated through the N-terminal cysteine thiol to keyhole limpet hemocyanin. The antigens were emulsified with complete Freund's adjuvant for the initial immunization and with incomplete Freund's adjuvant for booster injections. The immunogen emulsions were injected subcutaneously into New Zealand White rabbits, and antibody generation against the specific peptide was monitored by competitive ELISA. Antibodies present in serum from the last bleed were affinity-purified using a gel cross-linked with the appropriate synthetic phosphopeptide. The specificity of the antibodies was demonstrated in cells expressing melanopsin or melanopsin mutated in either Ser-381 or Ser-398 (see below). For detection of non-phospho-site-specific melanopsin in immunoblotting, a commercial antibody raised against a C-terminal peptide was used (abMel-WB) code no. PA1-781 (Thermo Fisher Scientific, Waltham, MA), whereas immunostaining was performed with an in-house-produced C-terminally directed rabbit anti-melanopsin antibody code no. 41K9 (abMel) (17).

##### Plasmids

The full coding sequence of rat melanopsin cDNA was amplified by PCR using Pfu polymerase (Agilent Technologies, Santa Clara, CA), AGCATGAACTCTCCTTCAGAA and CTACATCCTTCGGTCCAG as primers, and a previously described plasmid containing the cDNA of rat melanopsin as template (11). The obtained PCR fragment was cloned in pcDNA5/FRT/TO-TOPO (Invitrogen), and the resulting plasmid (prMel-21) harboring nucleotides 196–1623 of AY072689 was verified by sequencing (Eurofins MWG Operon, Ebersberg, Germany). The plasmid prMel-21 was used both to generate cells expressing WT-melanopsin (rMel-WT) and as the template for plasmids containing mutations in the codons for Ser-381 and Ser-398. The mutations resulting in Ser to Ala substitutions and thus preventing phosphorylation were done using QuikChange II site-directed mutagenesis kit (Agilent Technologies). The primer set CCACCCCTCCCTCGCCTACCGCTCTACC and GGTAGAGCGGTAGGCGAGGGAGGGGTGG and primer set CAGTCCTCAGACCTCGCCTGGATCTCTGGGC and GCCCAGAGATCCAGGCGAGGTCTGAGGACTG were used to generate the amino acid substitution at positions 381 (prMelS381A) and 398 (prMelS398A), respectively, the nucleotides changed are underlined. Generation of a plasmid carrying mutations in both codons (prMelS381 + 398A) was made using prMelS381A as template and the primer pair used for the S398A mutation. All plasmids were verified by sequencing (Eurofins MWG Operon).

##### Cell Culture

Flp-In T-Rex-293 cells (Invitrogen) derived from the human embryonic kidney cell line HEK-293 were used for transfection. Flp-In T-Rex-293 cells stably express a Tet repressor and contain a single integrated Flp recombination target (FRT) site. The cells were grown in DMEM (Invitrogen) supplemented with 10% tetracycline negative fetal calf serum (Invitrogen), 100 units/ml penicillin, and 0.1 mg/ml streptomycin (Biological Industries, Haemek, Israel/Invitrogen), 100 μg/ml zeocin (Invitrogen), and 15 μg/ml blasticidin (InvivoGen, San Diego, CA). The cells were co-transfected with one of the above described plasmids (prMel-21, prMelS381A, prMelS398A, or prMelS381 + 398A) and pOG44 (Invitrogen) expressing Flp recombinase (Invitrogen) according to the supplier's protocol. The day after transfection, cells were split, and the subsequent day 100 μg/ml hygromycin B (Invitrogen) as opposed to zeocin was added to the medium to select for Flp-mediated integration in the host cell genome. Due to the single FRT-site, the transfected cells are isogenic, rendering clonally selection unnecessary. The pcDNA5/FRT/TO-TOPO vector used for the melanopsin plasmids (prMel-21, prMelS381A, prMelS398A, or prMelS381 + 398A) contains a tetracycline-regulated promoter resulting tetracycline inducible expression of either WT melanopsin (rMel-WT) or melanopsin harboring single or double mutations rMel-S381 + 398A in the transfected cells.

For immunocytochemistry, 2 × 10^5^ cells were seeded on Lab-Tek^TM^ Permanox^TM^ Chamber Slides (Thermo Fisher Scientific), whereas ∼3 × 10^6^ cells were seeded in T25 flasks for immunoblots evaluating the time course of phosphorylation/dephosphorylation. The cells were either grown in the presence of tetracycline for induction of melanopsin or in absence of tetracycline. The latter served as negative controls. A tetracycline concentration-dependent increase in the expression of melanopsin was found, and 1 μg/ml tetracycline was optimal and chosen. The day after seeding, the medium was changed with medium containing 10 μm retinal (Sigma) and tetracycline. On the subsequent day the cells were either light-stimulated (white light >300 lux) or kept as dark controls. Cells for immunocytochemistry were fixated for 15 min in Stefanini's fixative and stored at −20 °C until further processing. After removal from the dark or light incubator, cells for immunoblotting were immediately placed on ice, washed twice in ice-cold phosphate-buffered saline (PBS), and extracted for 30 min on ice using Cell Extraction Buffer (Invitrogen) supplemented with 10 mm EDTA, Halt Phosphatase Inhibitor Mixture (Thermo Fisher Scientific), and Protease Inhibitor Mixture (Sigma). The whole-cell lysates were cleared by centrifugation for 10 min at 15,000 × *g*, 4 °C before storage at −80 °C.

##### Inhibition of Kinases

Kinase inhibitors from Merck Millipore (Copenhagen, Denmark) were dissolved in DMSO. The inhibitors (targeted kinases are in parentheses) and their concentrations were: 5 μm H-89 (PKA), 1 μm Gö 6850 (PKC), 0.1 mm βARK1 (β-adrenergic receptor kinase 1)/GRK2 (G protein receptor kinase 2), 25 μm D4476 (casein kinase 1), 10 μm PF-4708671 (ribosomal S6 kinase (S6K1)), 10 μm DG2 (S6K1), and 5 μm BI-D1870 (p90 ribosomal S6 kinases 1–4). 1.5 × 10^6^ cells were seeded in each well of 6-well plates, and individual inhibitors were added 30 min before light stimulation. Otherwise experiments were as described above. Three to five experiments were performed with each inhibitor, which also included both vehicle and non-vehicle controls. Induction of phosphorylation in the presence or absence of inhibitor was compared.

##### Animals and Tissue

24 Wistar male rats (Taconic, Ejby, Denmark) weighing 150–200 g housed with free access to food and water and under regulated temperatures entrained to 12 h light:12-h dark cycle (zeitgeber (ZT) 0, lights on; ZT12, lights off) were used. 12 rats were decapitated in dim red light at ZT24 and had their eyes removed. Another 12 rats were light-exposed for 5 min (>300 lux of white light) after which they were decapitated, and their eyes were removed. Between decapitation and eye removal the rat head was placed in wet ice (0 C°), and the eyes were removed within 10 min. One eye from each animal was dissected and immersion fixated in Stefanini's fixative over night. The other eye was dissected, and the anterior chamber was removed whereby the retina was fixed *in situ* in the eyecup in Stefanini's fixative overnight. The retina was then removed for flat mount immunohistochemistry and placed in cryoprotection until staining for melanopsin (see below) or the eye was cut in a cryostat (Leica Microsystems, Ballerup, Denmark) in sections of 12–14 μm and mounted on glass, frozen, and stored at −80 °C until processed for immunohistochemistry.

##### Immunoblotting

**FIGURE 2.**
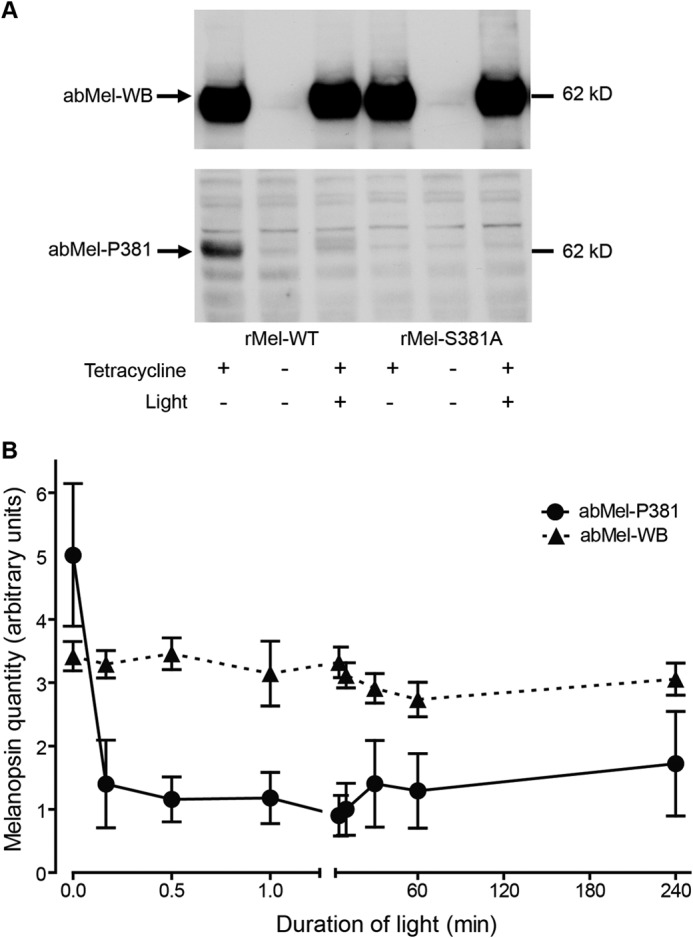
**Light-dependent changes in phosphorylation of melanopsin at Ser-381.** HEK-293 cells expressing native melanopsin (*rMel-WT*) or melanopsin mutated in Ser-381 (*rMel-S381A*) were kept in the dark or light-stimulated for different time periods. *A*, melanopsin expression was induced by growing the cells in the presence of tetracycline (1 μg/ml). The melanopsin expression in the dark or after 10 min of light stimulation was detected by immunoblotting on whole cell extracts using a non-phospho-site-specific melanopsin antibody (*abMel-WB*, *upper panel*) or an antibody against melanopsin phosphorylated at Ser-381 (*abMel-P381*, *lower panel*). Light dephosphorylates melanopsin (*rMel-WT*) at amino acid 381 as detected by the phospho-site-specific antibody, whereas melanopsin was undetectable using this antibody in cells expressing the Ser to Ala mutant (*rMel-S381A*). The position of a molecular weight marker is indicated to the *right. B*, shows the time course of light-induced dephosphorylation of melanopsin at Ser-381 in melanopsin-expressing cells (*rMel-WT*) exposed to light for time periods between 10 s and 4 h. Whole cell extracts were immunoblotted with the phospho-Ser-381 specific antibody (abMel-P381 ●) and the non-phospho-site-specific antibody (abMel-WB (▴). Quantifications of film exposures are shown as the means ± S.E.; *n* = 4. The expression of melanopsin did not vary when examined using abMel-WB, whereas the amount of melanopsin phosphorylated at Ser-381 decreased significantly by light exposures between 10 s and 4 h as compared with dark control.

SDS-PAGE and blotting were performed using NuPAGE electrophoresis and blotting systems (Invitrogen) as previously described (18). Melanopsin was detected using the following primary antibodies abMel-WB (1:5000), abMel-P381 (1:5000), and abMel-P398 (1:10000). Peroxidase-conjugated monoclonal mouse anti-rabbit IgG (1:5000, 211-032-171, Jackson ImmunoResearch, West Grove, PA) was used as a secondary antibody, and Pierce ECL Western blotting substrate (Thermo Fisher Scientific) was used for visualization. Quantification of exposures of immunoblots was done using ImageJ 1.49g (19). Digital images were taken using a Canon EOS 500D camera equipped with a Canon EF-S 60 mm 2.8 macro, and the “area” values reflecting both area and intensity were used to calculate the means ± S.E. depicted in Figs. 2, 4, and 6.

##### Immunostaining

Single and double antigen immunohistochemistry/cytochemistry were performed as described in detail previously (20). Briefly, non-phospho-site-specific melanopsin was visualized by our in-house rabbit anti-melanopsin antibody code no. 41K9 (abMel) and either an Alexa 488/594-conjugated donkey anti-rabbit antibody (code no: A-21206/21207, Molecular Probes, diluted 1:800) or a biotinylated donkey anti-rabbit (Fab)_2_ (code no. 711-066-152, Jackson ImmunoResearch Laboratories, West Grove, PA, diluted 1:800) in combination with avidin-biotin-peroxidase complex (ABC) (VWR International, Roedovre, Denmark) followed by biotinylated tyramide (Tyramide System Amplification, PerkinElmer Life Sciences) and streptavidin-Alexa 488 (code no. 016-488-084 Jackson ImmunoResearch Laboratories, diluted 1:500). The phospho-site-specific melanopsin antibody directed against the phosphorylated Ser in position 398 (abMel-P398) was visualized as either the melanopsin antibody abMel or when using two antibodies raised in the same species by the tyramide amplification system described above. The phospho-site-specific melanopsin antibody directed against the phosphorylated Ser in position 381 (abMel-P381) was visualized by EnVision^TM^ (code no. K4002, Dako, Copenhagen, Denmark, diluted 1:2) followed by Alexa Fluor® 488 tyramide (code no. AT20922, Molecular Probes, diluted 1:200). As a control the primary antibodies were omitted, which eliminated all staining. Furthermore, no melanopsin staining was found in retinal sections from melanopsin deficient mice (OPN4^−/−^) (21). The absence of staining of the mutant cell lines confirmed the specificity of the antibodies. Fluorescent images were obtained using an iMIC confocal microscope (Till Photonics, FEI, Munich, Germany) equipped with the appropriate filter settings for detecting CY2/Alexa 488 and Texas Red/Alexa 561/594. Images were adjusted for brightness and contrast in Adobe Photoshop CS5 and mounted into plates using Adobe Illustrator CS5.

##### Ca^2+^ Measurements

To elucidate the possible functional significance of Ser-381 and Ser-398, we performed [Ca^2+^]*_i_* measurements in HEK-293 cells expressing tetracycline-inducible melanopsin (rMel-WT) or the following melanopsin mutants: rMel-S381A, rMel-S398A, and rMel-S381 + S398A using the Ca^2+^-sensitive dye, Rhod-2 (Invitrogen). 24 h before experiments, cells were seeded on 1.76-cm^2^ HCl- and EtOH-washed coverslips in 28-cm^2^ Petri dishes in the absence or presence of 1 μg/ml tetracycline. Cells were incubated for 1 h in the dark with retinal (10 μm) and 5 μm Rhod-2 before measurements. From the addition of retinal, the cells were exclusively visualized in red light. After incubation, coverslips were mounted in a Warner RC-26G perfusion chamber and mounted on top of the iMIC microscope system (see below). Cells were continuously perfused with 37 °C Krebs solution (150 mm NaCl, 6 mm KCl, 1 mm MgCl_2_, 1.5 mm CaCl_2_, 10 mm HEPES, 10 mm glucose, pH 7.4 using NaOH) using a SH27A inline heater with TC324B temperature controller. To analyze melanopsin expression, cells remaining in the Petri dish were washed 2× in ice-cold PBS and collected in 150 μl of radioimmune precipitation assay buffer (150 mm NaCl, 1 mm EGTA, 1 mm NaVO_3_, 20 mm Tris-HCl, pH 7.5, 1% Nonidet P-40, 0.1% SDS, 1% sodium deoxycholate and Complete Mini protease inhibitor mixture) and stored at −20 °C. Harvested cells were lysed by sonication and cleared by centrifugation (20,000 × *g*, 5 min), and protein concentration was measured by DC assay (Bio-Rad). Melanopsin expression was analyzed by immunoblotting as described above using the non-phospho-site-specific antibody (abMel-WB). There was no significant difference in the expression levels between WT and the various mutants (data not shown). Fluorescent signals were visualized using an Ixon 885 camera (Andor, Belfast, N. Ireland) through a 40× oil immersion objective (Olympus, Tokyo, Japan) on an iMIC2000 inverted microscope with Polychrome V monochromator (Till Photonics, Gräfelfing, Germany). Microscope control, signal visualization (binning 2 × 2), and analysis were performed in Live Acquisition software (Till Photonics). Rhod-2 signals of >590 nm were visualized through a 49005-ET filterset (Chroma Technology, Bellows Falls, VT) every 10 s after 50 ms of 550-nm (bandwidth 10 nm) excitation at low intensity 0.005 watt/cm^2^. Melanopsin was blue light stimulated using an RL5-B2545 5-mm LED (Superbright LEDs, St. Louis, MO) with an intensity of 0.16 watt/cm^2^. Cross-talk between stimulation diode and Rhod-2 excitation was very limited. Autofluorescence in non-loaded cells was subtracted from all signals, and these were further linearly corrected for signal rundown.

##### Data Analysis

Quantitative data are presented as the means ± S.E. One-way analysis of variance followed by Bonferroni's multiple comparison test was used for statistical analysis. Calculations and graphs were made using GraphPad Prism software (Version 4.0, San Diego, CA). *p* < 0.05 was considered statistically significant.

## RESULTS

### 

#### 

##### Light Dephosphorylates Melanopsin at Ser-381

**FIGURE 3.**
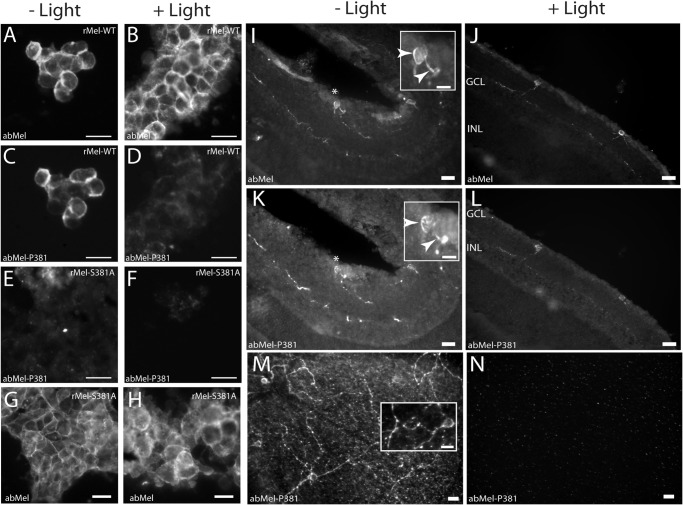
**Photomicrographs showing light-induced changes in phosphorylation of melanopsin at Ser-381 *in vitro* and *in vivo*.** *A–H*, HEK-293 cells expressing tetracycline-induced native melanopsin (*rMel-WT*) (*A–D*) and melanopsin mutated in Ser-381 (*rMel-S381A*) (*E–H*) were kept in the dark (*A*, *C*, *E*, and *G*) or light-stimulated (*B*, *D*, *F*, and *H*) for 5 min. The cells were double-immunostained for melanopsin using a non-phospho-site-specific antibody (*abMel*) (see *A* and *B* and see *G* and *H*) and an antibody against melanopsin phosphorylated at Ser-381 (*abMel-P381*) (*C–F*). Melanopsin is localized to the cell membrane, and during dark melanopsin is phosphorylated in Ser-381 (*C*). Light stimulation causes a marked decrease in staining using the phospho-site-specific antibody (*D*). Melanopsin phosphorylation was absent in cells expressing the Ser to Ala mutant at position 381 (*E* and *F*), whereas staining using the non-phospho-site-specific antibody (*abMel*) was unaffected by mutation at Ser-381 (*G* and *H*). Wistar rats were kept in 12:12 h light/dark cycle and sacrificed in dim red light at ZT24 (*I*, *K*, and *M*) or 5 min after light was turned on (*J*, *L*, and *N*). Cross-sections of retina were double immunostained for melanopsin with the non-phospho-site-specific antibody (*abMel*) (*I* and *J*) and the antibody against melanopsin phosphorylated at Ser-381 (abMel-P381) (*K–L*). Flat mounts of retina were also stained with the phospho-site-specific antibody (*M–N*). In dark-adapted retina melanopsin phosphorylated at Ser-381 was visible in both sections (*K*) and flat mounts (*M*) and found to be co-stored in all cells identified by the non-phospho-site-specific melanopsin antibody (*I*). After 5 min of light exposure melanopsin phosphorylated in position Ser-381 was almost completely eliminated (*L* and *N*), whereas melanopsin in the same section was unaffected (*J*). *Insets* in *I* and *K*, high power magnification of the cell indicated by *asterisks* illustrating melanopsin in the cell membrane using the non- and the phospho-site-specific antibody (*arrowheads*). *Inset M*, high power magnification showing that melanopsin phosphorylated at Ser-381 is located in the membrane and processes of the cell. *Scale bars*: *A–F*, 25 μm; *G* and *H*, 15 μm; *I–L*, 30 μm (*inset*, 15 μm); *M* and *N*, 15 μm (*inset*, 15 μm). *GCL*, ganglion cell layer; *INL*, inner nuclear layer.

Because melanopsin is activated by light, we examined whether exposure to light or dark caused changes in phosphorylation at Ser-381 or Ser-398 in rat melanopsin. Immunoblotting of protein extracts from melanopsin-expressing HEK-293 cells using the phospho-site-specific antibody against Ser-381 demonstrated that melanopsin during dark was phosphorylated at this position and that no immunoreactive band was detectable in cells expressing melanopsin in which Ser-381 was substituted with Ala (Fig. 2*A*, *lower panel*). As revealed by immunoblotting, exposure of the melanopsin-expressing HEK-293 cells to light caused a rapid (within 10 s) and significant dephosphorylation at Ser-381 that persisted throughout the 4-h examination period (Fig. 2*B*). Melanopsin detected using the non-phospho-site-specific antibody was unchanged during light exposure (Fig. 2, *A*, *upper panel*, and *B*). In melanopsin-expressing HEK-293 cells kept in the dark, strong immunostaining of the cell membrane was observed with both the non-phospho-site-specific antibody and the Ser-381 phospho-specific antibody (Fig. 3, *A* and *C*). After 5 min of light exposure the immunostaining with the Ser-381 phospho-site-specific antibody had almost disappeared (Fig. 3*D*), whereas the staining pattern with the non-phospho-site-specific antibody was unchanged (Fig. 3*B*). When Ser at position 381 was replaced by Ala, no immunostaining with the phospho-site-specific antibody was detectable (Fig. 3, *E* and *F*), whereas staining with the non-phospho-site-specific antibody was unaffected by the mutation (Fig. 3, *G* and *H*). Immunohistochemical data obtained *in vivo* using the two antibodies for staining of ipRGCs in the rat retina showed a similar light response. Thus in dark-adapted rat retina identical melanopsin staining was present in cell bodies and processes with both the phospho-381-specific and the non-phospho-site-specific antibody (Fig. 3, *I*, *K*, and *M*). After 5 min of light stimulation of the rat, staining for melanopsin phosphorylated at position 381 was markedly reduced (Fig. 3, *L* and *N*), whereas melanopsin visualized with the non-phospho-site-specific antibody was unaffected (Fig. 3*J*).

##### Light Phosphorylates Melanopsin at Ser-398

**FIGURE 4.**
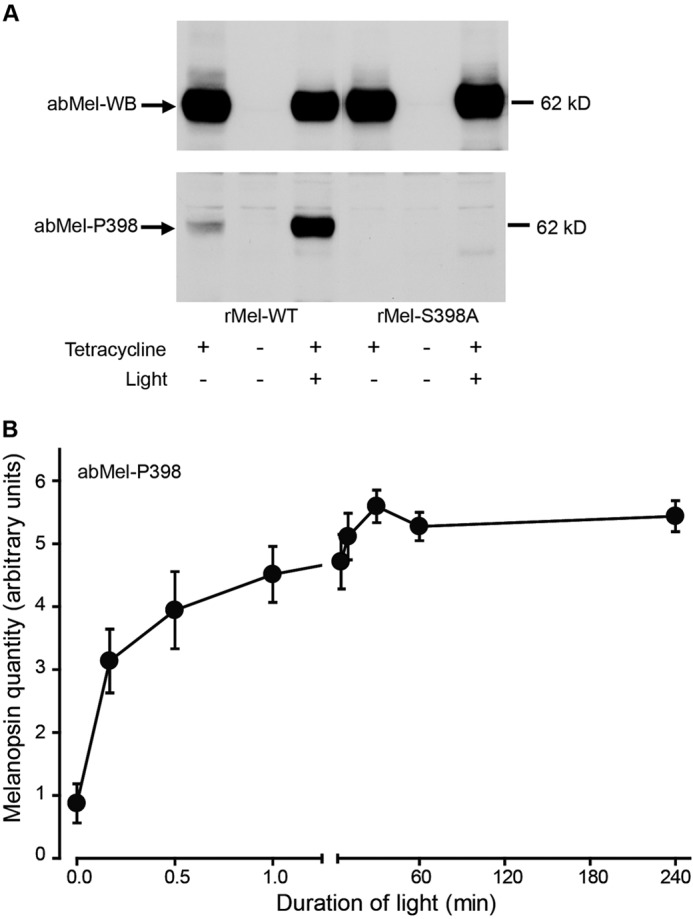
**Light-dependent changes in phosphorylation of melanopsin at Ser-398.** HEK-293 cells expressing tetracycline-induced native melanopsin (*rMel-WT*) or melanopsin mutated in Ser-398 (*rMel-S398A*) were kept in the dark or light-stimulated for different time periods. *A*, the melanopsin expression in cells kept in the dark or after 10 min of light stimulation were detected by immunoblotting on protein extracts using a non-phospho-site-specific antibody (*abMel-WB*, *upper panel*) or an antibody against melanopsin phosphorylated at Ser-398 (*abMel-P398*, *lower panel*). Light phosphorylates melanopsin (*rMel-WT*) at Ser-398, a response that is annulled in cells expressing the serine to alanine mutant (rMel-S398A) and, therefore, not detected by the phospho-site-specific antibody. The position of a molecular weight marker is indicated to the *right. B* shows the time course of light-induced phosphorylation of melanopsin at Ser-398 in melanopsin-expressing cells (*rMel-WT*) exposed to light for time periods between 10 s and 4 h. Whole cell extracts were immunoblotted with the phospho-Ser-398-specific antibody (*abMel-P398*) and the non-phospho-site-specific antiserum (*abMel-WB*, see Fig. 2*B*). Quantifications of film exposures are shown as the means ± S.E.; *n* = 4. The expression of melanopsin phosphorylated at Ser-398 increases significantly by light exposures between 10 s and 4 h as compared with dark control.

**FIGURE 5.**
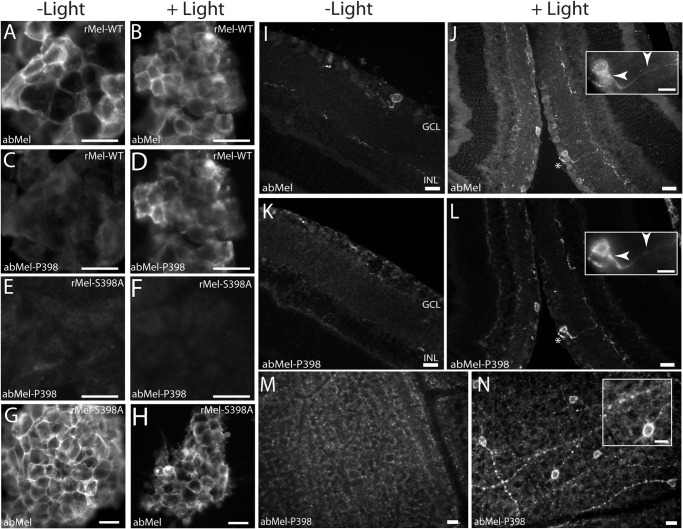
**Photomicrographs showing light-induced changes in phosphorylation of melanopsin at Ser-398 *in vitro* and *in vivo*.** *A–H*, HEK-293 cells expressing tetracycline-induced native melanopsin (*rMel-WT*) (*A–D*) and melanopsin mutated at Ser-398 (*rMel-S398A*) (*E–H*) were kept in the dark (*A*, *C*, *E*, and *G*) or light-stimulated (*B*, *D*, *F*, and *H*) for 5 min. The cells were double immunostained for melanopsin using a non-phospho-site-specific antibody (*abMel*) (see *A* and *B* and see *G* and *H*) and an antibody against melanopsin phosphorylated at Ser-398 (*abMel-P398*) (*C–F*). During darkness melanopsin is unphosphorylated at Ser-398 (*C*), whereas light stimulation induces a marked phosphorylation of melanopsin at this site (*D*). Light-induced melanopsin phosphorylation was absent in cells expressing the mutant Ser to Ala at position 398 (*E* and *F*), whereas staining using the non-phospho-site-specific antibody (*abMel*) was unaffected by mutation at Ser-398 (*G* and *H*). *I–N*, Wistar rats were kept in 12:12 h light/dark cycle and killed in dim red light at ZT24 (*I*, *K*, and *M*) or 5 min after light was turned on (*J*, *L*, and *N*). Cross-sections of retina were double-immunostained for melanopsin with a non-phospho-site-specific antibody (*abMel*) (*I* and *J*) and the antibody against melanopsin phosphorylated at Ser-398 (*abMel-P398*) (*K–L*). Flat mounts of retina were also stained with the phospho-site-specific antibody (*M* and *N*). During darkness no immunostaining was observed using the antibody against melanopsin phosphorylated at Ser-398 (*K* and *M*). 5 min after light was turned on melanopsin phosphorylated at position Ser-398 was visible in both retinal sections (*L*) and flat mount retina (*N*) and found to be co-stored in all cells identified by the non-phospho-site-specific melanopsin antiserum (*J*). *Insets* in *J* and *L*, high power magnification of the cell indicated by *asterisks* illustrating melanopsin in the cell membrane using the non- and the phospho-site-specific antibody (*arrowheads*). *Inset* in *N*, high power magnification showing that melanopsin phosphorylated at Ser-398 is located in the membrane and processes of the cell. *Scale bars*, *A* and *C*, 20 μm; *B* and *D*, 30 μm; *E–H*, 20 μm; *I–L*, 25 μm (*inset*, 15 μm); *M* and *N*, 15 μm (*inset*, 15 μm). *GCL*, ganglion cell layer; *INL*, inner nuclear layer.

Immunoblotting of protein extracts from HEK-293 cells expressing WT melanopsin with the antibody against melanopsin phosphorylated at Ser-398 disclosed that light provoked phosphorylation at this position, whereas it was unphosphorylated during dark (Fig. 4*A*, *lower panel*). The light-induced phosphorylation at Ser-398 was annulled in cells expressing the mutant Ser to Ala at position 398 and was thus undetected by the Ser-398 phospho-site-specific antibody (Fig. 4*A*, *lower panel*). The light-induced phosphorylation of melanopsin at Ser-398 in the HEK-293 cells occurred within the first minute and persisted throughout the 4-h examination period (Fig. 4*B*). In accordance, immunostaining of the cells with the antibody against melanopsin phosphorylated at Ser-398 detected no signal during dark (Fig. 5*C*), but it showed strong immunostaining of the cell membrane after 5 min light (Fig. 5*D*) with a localization identical to that visualized with the non-phospho-site-specific antibody (Fig. 5, *A* and *B*). The immunostaining with the phospho-site-specific antibody after light was eliminated in cells expressing melanopsin mutated at Ser-398 (Fig. 5, *E* and *F*), whereas staining with the non-phospho-site-specific antibody was unaffected by the mutation (Fig. 5, *G* and *H*). Also, no change was detected with the non-phospho-site-specific antibody in ipRGCs of rats exposed to light (Fig. 5, *I* and *J*), whereas a similar induction of phosphorylation of melanopsin at Ser-398 was observed (Fig. 5, *K–N*).

##### Phosphorylation at Ser-381 and Ser-398 Is Independent of Each Other

**FIGURE 6.**
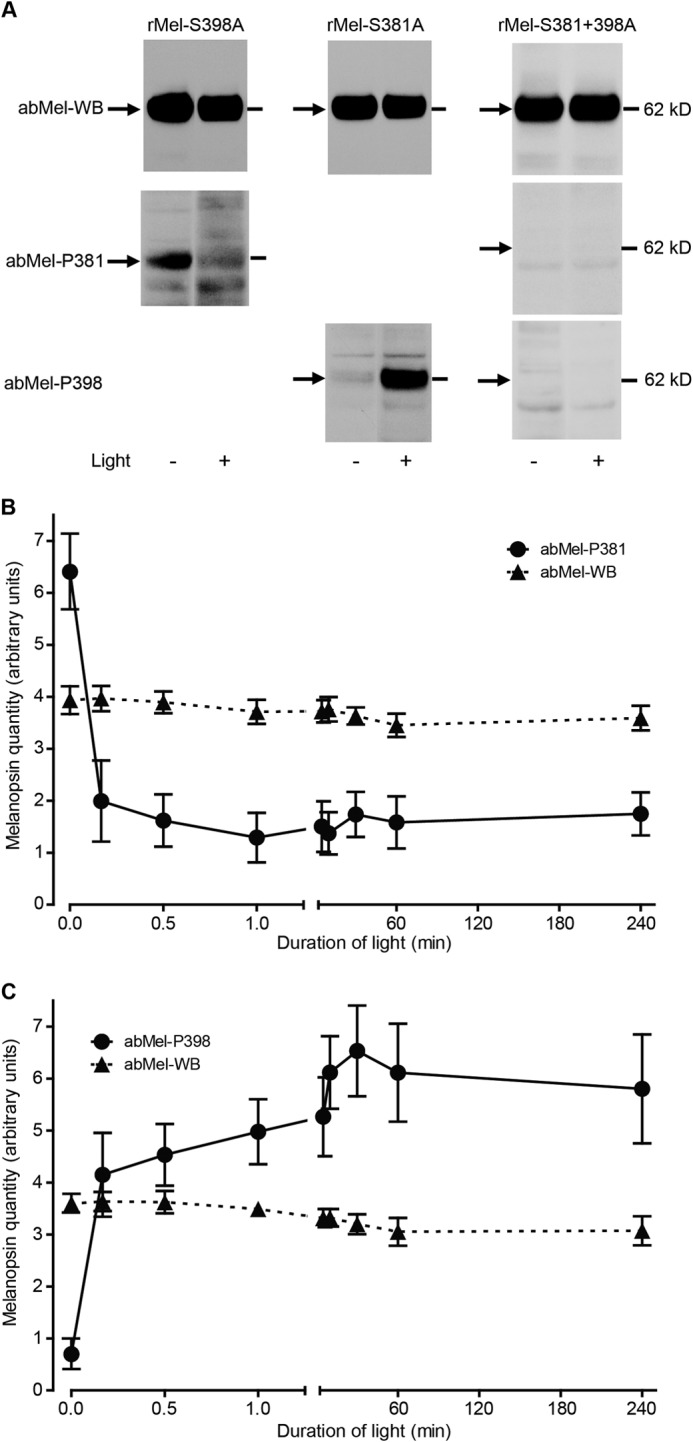
**Light-dependent changes in phosphorylation of melanopsin at Ser-381 and Ser-398 are independent.** HEK-293 cells expressing tetracycline-induced melanopsin mutated in either Ser-381 (rMel-S381A), Ser-398 (rMel-S398A), or both (*rMel-S381* + *398A*) were kept in the dark or light stimulated for different time periods. *A*, melanopsin expression in the dark or after 10 min of light stimulation was detected by immunoblotting on whole cell extracts using a non-phospho-site-specific antibody (*abMel-WB*, *upper panels*) or antibodies against either melanopsin phosphorylated at Ser-381 (*abMel-P381*, *middle panels*), or Ser-398 (*abMel-P398*, *lower panels*). Light dephosphorylates melanopsin at Ser-381 irrespective of the mutation at Ser-398 (*rMel-S398A*) (*left panels*), and light phosphorylates melanopsin at Ser-398 irrespective of the mutation at Ser-381 (rMel-S381A) (*center panels*). Blots of extracts of cells expressing double-mutated melanopsin (*rMel-S381* + *298A*) are shown in the *right panels*. The antibodies used for immunoblots are indicated to the *left*, and the position of a molecular weight marker is indicated to the *right. B*, shows the time course of light-induced dephosphorylation of melanopsin at Ser-381 in melanopsin-expressing cells mutated at Ser-398 (rMel-S398A) exposed to light for time periods between 10 s and 4 h. Whole cell extracts were immunoblotted with the phospho-Ser-381-specific antibody (*abMel-P381* (●)) and the non-phospho-site-specific antibody (*abMel-WB* (trif)). Quantifications of film exposures are shown as the means ± S.E.; *n* = 4. The expression of melanopsin phosphorylated at Ser-381 decreased significantly by light exposures between 10 s and 4 h as compared with the dark control. No changes were found in melanopsin expression evaluated using the non-phospho-site-specific antibody (*abMel-WB*). *C*, shows the time course of light-induced phosphorylation of melanopsin at Ser-398 in melanopsin-expressing cells mutated at Ser-381 (rMel-S381A) exposed to light for time periods between 10 s and 4 h. Whole cell extracts were immunoblotted with the phospho-Ser-398-specific antibody (abMel-P398 (●)) and the non-phospho-site-specific antibody (*abMel-WB* (▴)). Quantifications of film exposures are shown as the means ± S.E.; *n* = 4. The expression of melanopsin phosphorylated at Ser-398 increased significantly by light exposures between 10 s and 4 h as compared with the dark control. No changes were found in melanopsin expression evaluated using the non-phospho-site-specific antibody (*abMel-WB*).

**FIGURE 7.**
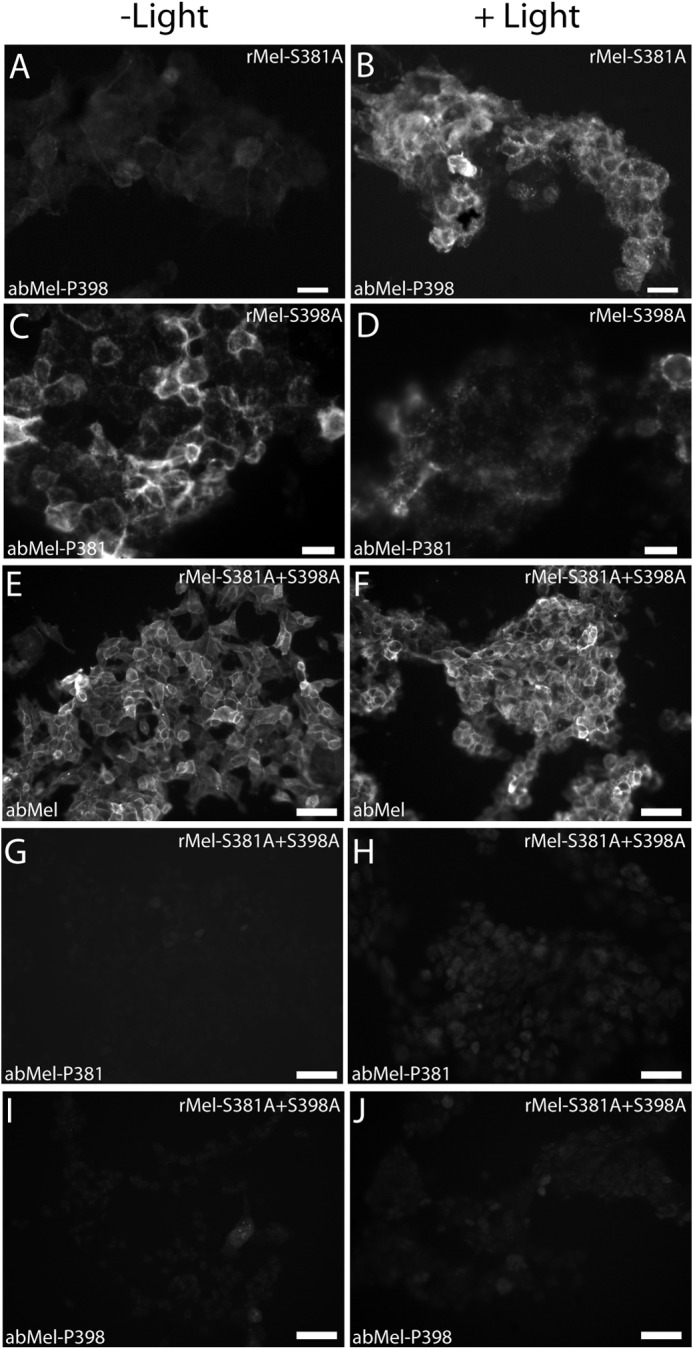
**Photomicrographs showing immunostaining for melanopsin and phosphorylated melanopsin in mutant-expressing cells.** HEK-293 cells expressing tetracycline-induced melanopsin mutated at either Ser-381 (*rMel-S381A*) (*A* and *B*), Ser-398 (*rMel-S398A*) (*C* and *D*), or both amino acids (*rMel-S381A* + *S398A*) (*E–J*) were kept in the dark (*A*, *C*, *E*, *G*, and *I*) or light-stimulated (*B*, *D*, *F*, *H*, and *J*) for 5 min. The cells were immunostained using the non-phospho-site-specific antibody (abMel) (*E* and *F*) or antibodies against melanopsin phosphorylated at Ser-381 (*abMel-P381*) (see *C* and *D* and see *G* and *H*) or at Ser-398 (*abMel-S398*) (see *A* and *B* and see *I* and *J*). Mutation at Ser-381 did not affect the light-induced changes observed using the antibody against melanopsin phosphorylated at Ser-398 (*abMel-P398*) (*A* and *B*) or vice versa (*C* and *D*). No immunostaining was detected in cells expressing melanopsin mutated at both Ser-381+ Ser-398 using the phospho-site-specific antibodies abMel-P381 (*G* and *H*) or abMel-P398 (*I* and *J*), whereas staining with the non-phospho-site-specific antibody (abMel) was unaffected by the double mutation (*E* and *F*). *Scale bars*: *A–D*, 20 μm; *E–J*, 50 μm.

To examine whether elimination of phosphorylation at Ser-381 influences the light/dark-dependent phosphorylation at Ser-398 and vice versa, we examined phosphorylation of cells expressing melanopsin mutated at either Ser-381 or Ser-398. Immunoblotting of protein extracts of cells expressing the melanopsin mutant Ser to Ala at position 398 with the Ser-381 phospho-site-specific antibody showed phosphorylation during dark and a rapid light-induced dephosphorylation (Fig. 6, *A*, *left panels*, and *B*), findings that were identical with to those observed in non-mutated melanopsin (Fig. 2). Accordingly, the cells mutated at Ser-398 were strongly immunostained for phospho-381-melanopsin during dark, a staining that almost disappeared after 5 min of light (Fig. 7, *C* and *D*). Examination of cells expressing the melanopsin mutant Ser to Ala at position 381 with the antibody against melanopsin phosphorylated at Ser-398 revealed a light-induced phosphorylation similar to native melanopsin as evaluated both by immunoblotting (Fig. 6, *A*, *center panels* and *C*) and by immunocytochemical staining (Fig. 7, *A* and *B*). As expected, no phosphorylation was detected at either Ser-381 or Ser-398 in cells expressing melanopsin mutated at both Ser-381 and Ser-398 by immunoblotting (Fig. 6*A*, *right panels*) and immunocytochemistry (Fig. 7, *G–J*). In the double mutants, however, a melanopsin band and membrane staining were demonstrated with the non-phospho-site-specific antibody (Figs. 6*A*, *right panels*, and 7, *E* and *F*).

##### PKC and S6K1 Seem to Be Involved in Phosphorylation at Ser-381 and Ser-398, Respectively

To obtain information about the kinases responsible for phosphorylation of melanopsin at Ser-381 and Ser-398, we performed experiments in the presence of various kinase inhibitors. A statistically significant 50% reduction in the increase of phosphorylation at Ser-381 when going from light to dark was observed in the presence of the PKC inhibitor Gö 6850. No effect on phosphorylation at Ser-381 was observed with inhibitors of PKA, GRK2, casein kinase 1, S6K1, or p90 ribosomal S6 kinases 1–4. The addition of either of the two S6K1 inhibitors (DG2 and PF-4708671) reduced the light-induced phosphorylation at Ser-398 to ∼70% compared with that of control cells. Although the reductions did not reach statistical significance, decreased phosphorylation was obtained with two independent inhibitors. Phosphorylation at Ser-398 was not inhibited by any of the other kinase inhibitors used.

##### Mutation of Ser-381 and Ser-398 in Melanopsin Decreases Light-induced Ca^2+^ Response

**FIGURE 8.**
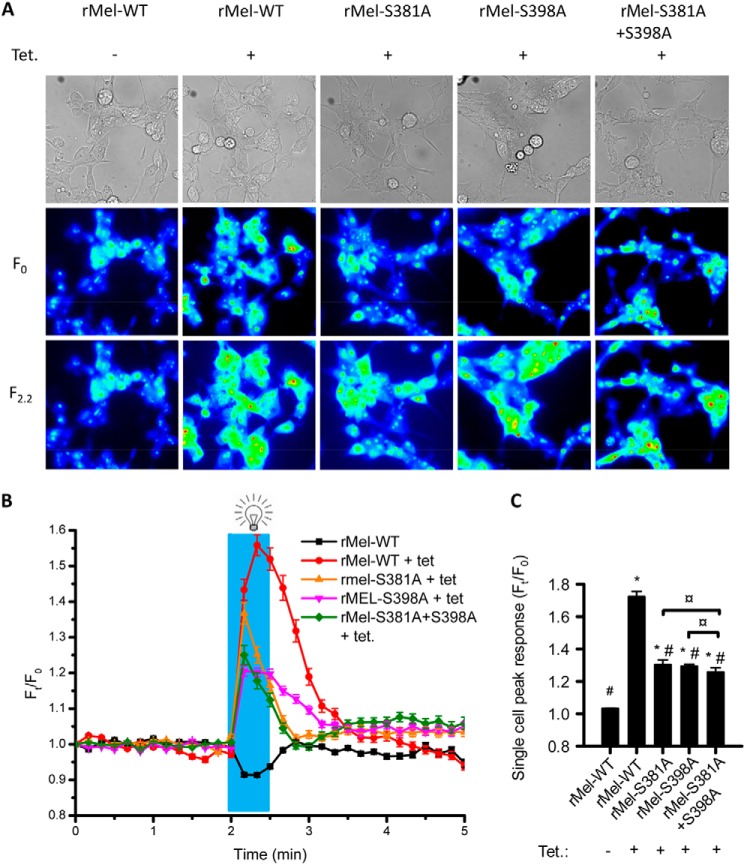
**Rhod-2 [Ca^2+^[rsqb]*_i_* imaging in melanopsin-expressing HEK-293 cells.** Cells were incubated with retinal and Rhod-2 in the dark for 1 h before measurements. *A*, representative images of HEK-293 cells with tetracycline-inducible melanopsin expression. Cell clone and the presence of tetracycline (*Tet*) in growth medium are indicated at the *top. Top images* are red light transluminescence images. The *two lower image panels* are Rhod-2 fluorescence intensity images before and 20 s into light exposure. *B*, average fluorescent intensity as function of time summarized for 114–223 individual cells in three independent experiments. The *blue bar* represents the time of light stimulation. *C*, because timing for maximal [Ca^2+^]*_i_* response differed between individual cells, peak [Ca^2+^]*_i_* response after light stimulation was calculated for individual cells, averaged, and summarized in *C*. Cell clone and 24 h exposure to tetracycline are indicated below. Data are presented as the means ± S.E., and statistical significance was evaluated by use of Mann-Whitney *U* test. Symbols represent statistical difference from: rMel-WT without tetracycline (*) and rMel-WT with tetracycline (# and ¤) between indicated cell clones.

To investigate if mutation of Ser-381 and Ser-398 in melanopsin affects light-induced Ca^2+^ signaling, we performed [Ca^2+^]*_i_* measurements in HEK-293 cells expressing WT melanopsin or the mutants rMel-S381A and rMel-S398A using the Ca^2+^-sensitive dye, Rhod-2. Emission spectrum for light stimulus did not overlap with the excitation spectrum for Rhod-2 (data not shown). In HEK-293 cells expressing rat melanopsin, we observed a fast and robust increase in cytosolic Ca^2+^ when cells were light-exposed as opposed to cells without melanopsin expression in which no Ca^2+^ response was observed (Fig. 8). All cells, however, gave robust responses to thrombin as a positive control for G protein-coupled signaling (not shown). Light-induced Ca^2+^ response was significantly reduced in cells expressing melanopsin mutated in Ser-381. Analysis of single cell responses further showed a significantly reduced time to peak response as compared with rMel-WT. Similarly, cells expressing melanopsin mutated in Ser-398 had significantly reduced Ca^2+^ responses to light stimulus. However, the kinetics of the Ser-398 mutant response was not significantly different from the one observed in cells expressing WT melanopsin. To investigate if the effects of Ser-381 and Ser-398 mutation are combinatorial, we measured the light-induced Ca^2+^ response in cells expressing melanopsin carrying both mutations. Analyzing the response from single cells revealed a significantly reduced peak response in comparison to both WT melanopsin and the two individual mutants (Fig. 8*C*). Furthermore, the time from stimulus initiation to peak response was significantly reduced as compared with the response in WT melanopsin-expressing cells (data not shown).

## DISCUSSION

The predicted secondary structure of melanopsin indicates a long cytoplasmatic tail with many potential phosphorylation sites suggesting that this effector domain of melanopsin could be highly regulated, and recently a phosphorylation cassette was identified in mouse and zebrafish melanopsin (15). It was shown that this cassette is essential for signal deactivation from light stimulation, and it was suggested that a variation in phosphorylation sites provides flexibility in light-stimulated signaling. Using the bioinformatics web tool NetPhos 2.0, we identified a number of amino acids in the C-terminal tail of melanopsin with high probability of being phosphorylated, and of these, Ser-381 and Ser-398 were highly conserved in vertebrate melanopsin. Albeit the described deactivation cassette (Ser-386 to Ser-396) is conserved in rat melanopsin, neither Ser-381 nor Ser-398 is part of this cassette, and their phosphorylation has not previously been examined. We subsequently generated antibodies against melanopsin phosphorylated at Ser-381 and Ser-398. The specificity of the two phospho-site-specific antibodies was confirmed by immunoblotting and immunocytochemical staining of HEK-293 cells expressing melanopsin mutated in Ser-381 or in Ser-398, respectively. Using the antibody that recognizes phospho-Ser-381-melanopsin, we demonstrated that melanopsin both *in vitro* and *in vivo* is phosphorylated in this position during dark and is dephosphorylated when light is turned on. We consider the finding to be a genuine dephosphorylation at Ser-381. Another interpretation is however possible, namely that a light-induced phosphorylation of the nearby Ser-379 could disrupt the antibody binding, but the Ser-379 is not conserved among either mammalian or vertebrate species. In contrast to Ser-381, melanopsin at Ser-398 was unphosphorylated in dark and became phosphorylated after light stimulation. The latter finding is in accord with a previous study in which a general anti-phosphoserine antibody was used to provide evidence that melanopsin is phosphorylated in the C-terminal tail after 10 min of light exposure (14). It is interesting that different melanopsin phosphorylation sites are regulated by light in opposite directions, but similar findings have previously been reported, for example in light-dependent phosphorylation of *Drosophila* TRPC (22). It is likely that separate kinases as well as phosphatases become recruited and activated in either light or dark.

The changes in phosphorylation status at both Ser-381 and Ser-398 when light was turned on were rapid and lasted throughout the 4-h experimental period. The light-induced dephosphorylation of Ser-381 seemed to be somewhat faster than the phosphorylation at Ser-398, which may pose the question of whether the phosphorylation of Ser-398 depends on prior dephosphorylation of Ser-381. We address this question by examining cells in which the individual phosphorylation sites were eliminated by mutation and clearly showed that changes in phosphorylation at Ser-381 and at Ser-398 were independent of each other.

The activity of G protein-coupled receptors is often rapidly attenuated after activation typically accomplished by phosphorylation on the C-terminal tail by members of the GRK family of serine/threonine kinases (23). A possible role for GRKs in the overall phosphorylation of the C-terminal tail of mouse melanopsin after light stimulation has previously been addressed (14). Using co-immunoprecipitation assays, the Ca^2+^ response in cells treated with siRNA directed against GRKs, and double immunostaining, some evidence was provided that GRK2 could be an endogenous kinase in melanopsin phosphorylation (14). We did not, however, find any effect on phosphorylation of either Ser-381 or Ser-398 of the GRK2 inhibitor used in our kinase inhibitor experiments. This finding was further supported by our preliminary experiments using siRNA against GRK2 and GRK3 in which no effect on the phosphorylation of Ser-398 during light exposure was observed. It remains to be fully clarified which phosphatases and kinases are involved in the light-provoked dephosphorylation of Ser-381 and phosphorylation of Ser-398 in melanopsin. A search in the program group-based prediction system (GPS 2.1) indicates that both Ser-381 and Ser-398 are potential sites for the PKA, - C, and - G family, and Ser-398 in addition is a potential site for S6K (24). Using protein kinase inhibitors, we revealed that PKC could be at least partly responsible for phosphorylation of Ser-381 during dark, whereas light-induced phosphorylation of Ser-398 was decreased by two different inhibitors of S6K1.

When expressed in HEK-293 cells, all melanopsin constructs mediated robust light-induced elevations in intracellular Ca^2+^, albeit the Ca^2+^ response was significantly reduced in cells expressing melanopsin mutated at Ser residues. Kumbalasiri (7) *et al.* previously showed that Ca^2+^ responses in HEK-293 made photosensitive by melanopsin expression is exclusively mediated by Ca^2+^ release from intracellular stores. This may not be representative of the Ca^2+^ signaling in ipRGCs in which Ca^2+^ from intracellular stores is not released during photostimulation (6, 25). In these cells >90% of the observed Ca^2+^ response could be accounted for by influx via voltage-gated Ca^2+^ channels with the remaining 10% associated with melanopsin associated activation of TRPC7 (6). However, the melanopsin activation pathway seems to be the same in both cell types. G protein phospholipase C activation is associated with photosensation in both HEK-293 (8) cells and ipRGCs (26), leading to the cytosolic accumulation of diacylglycerol and inositol 1,4,5-triphosphate. In HEK-293 cells this mediates activation of the inositol 1,4,5-triphosphate receptor followed by Ca^2+^ release from intracellular stores, whereas in ipRGCs diacylglycerol accumulation activates TRPC7, which in turn depolarizes the membrane and activates voltage-gated calcium channels. Hence, store-operated Ca^2+^ release in melanopsin-expressing HEK-293 cells is a representative measure for melanopsin photoactivation in ipRGCs.

In a previous study examining light-induced kinetics of Ca^2+^ in HEK-293 cells expressing mouse melanopsin, phosphorylation of a cluster of serines and threonines in the region amino acid 386–396 was identified to control the deactivation response (15). No effect on the deactivation response was found when each of six phosphorylatable sites was mutated individually (15). Here we examined the role of phosphorylation at two single sites outside this domain in rat melanopsin on the intracellular Ca^2+^ response. We found that both the Ser-381 and Ser-398 mutation facilitated a reduced Ca^2+^ response when compared with WT melanopsin-expressing cells without showing reduced melanopsin protein expression. This is in contrast to previous studies in which C-terminal phosphorylation of mouse melanopsin was correlated to reduced G protein activation (14, 15), in line with the general phosphorylation-dependent desensitization of G protein-coupled receptors (27). Certainly, neither Ser-381 nor Ser-398 mutants showed a reduced desensitization. To the contrary, Ser-381 mutant and the double mutant Ser-381/Ser-398, both, showed a significantly shortened response peaking significantly before WT melanopsin. Hence, phosphorylation of Ser-381 seemingly protects melanopsin from desensitization. With regard to Ser-398, Blasic *et al.* (15) did not observe any significant effects on stimulated Ca^2+^ signaling when investigating melanopsin truncated after amino acid 396. However, data were normalized to the peak value to investigate deactivation kinetics, and it is, therefore, not possible to see whether this truncation affected melanopsin activation (15). In our study mutation of Ser-398 did not reveal any kinetic differences to WT melanopsin on top of the peak value and is most likely involved directly in melanopsin activation. Importantly, the combined effect of the double mutant shows the two phosphorylation mechanisms are independent. Although the phosphorylation events at the two sites seem not to be interdependent, it is possible that they are linked in terms of function. In the two single mutants, the effect on the Ca^2+^ response was in both cases less than in control cells, thus removing the basal phosphorylation at Ser-381, and removing light-induced phosphorylation at Ser-398 had the same effect. Furthermore, in the double mutant the effect on the Ca^2+^ response did not seem to be additive, which could indicate that both phosphorylation events are linked to the same mechanism of Ca^2+^ regulation.

## Footnotes

2 The abbreviations used are:

ipRGC: intrinsically photosensitive ganglion cell
FRT: Flp recombination target
GRK: G protein-coupled receptor kinase
S6K: ribosomal S6 kinase
TRPC: transient receptor potential ion channel
S6K1: ribosomal S6 kinase
ZT: zeitgeber.
